# Supplementary material for: Acidic CO2-to-HCOOH electrolysis with industrial-level current on phase engineered tin sulfide
Source: Nat Commun. 2023 May 18;14:2843. doi: 10.1038/s41467-023-38497-3 (PMC10195825; doi:10.1038/s41467-023-38497-3)
Supplement: Supplementary file 1 — Supplementary Information [file 41467_2023_38497_MOESM1_ESM.pdf]

Supplementary Information for

Acidic CO_2-to-HCOOH electrolysis with industrial-level current on phase engineered tin sulfide

Haifeng Shen[1,3], Huanyu Jin[1,3], Haobo Li[1,3], Herui Wang[2], Jingjing Duan[2], Yan Jiao[1], Shi-Zhang Qiao[1,*]

[1] School of Chemical Engineering, The University of Adelaide, Adelaide, SA 5005, Australia

[2] School of Energy and Power Engineering, Nanjing University of Science and Technology, Nanjing 210094, China

[3] These authors contributed equally: Haifeng Shen, Huanyu Jin, Haobo Li.

[*] Correspondence to E–mail: s.qiao@adelaide.edu.au

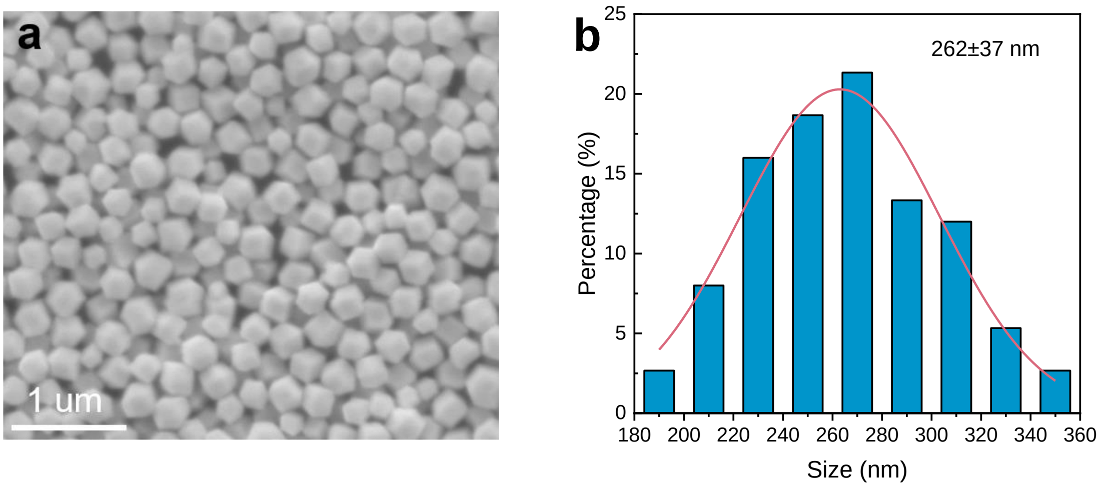

Supplementary Fig. 1 | a, FESEM image of π-SnS. **b**, Corresponding side length histogram.

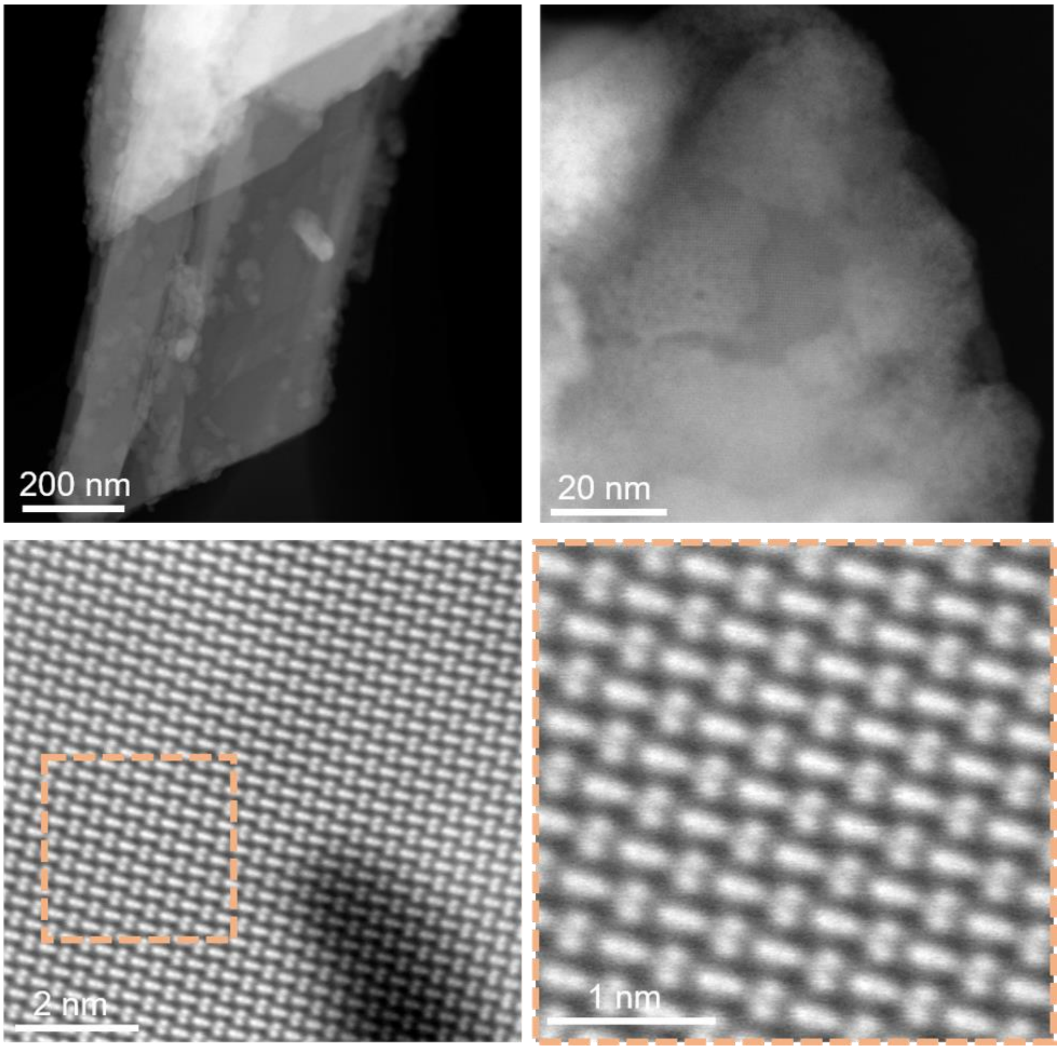

Supplementary Fig. 2 | HAADF-STEM images of α-SnS.

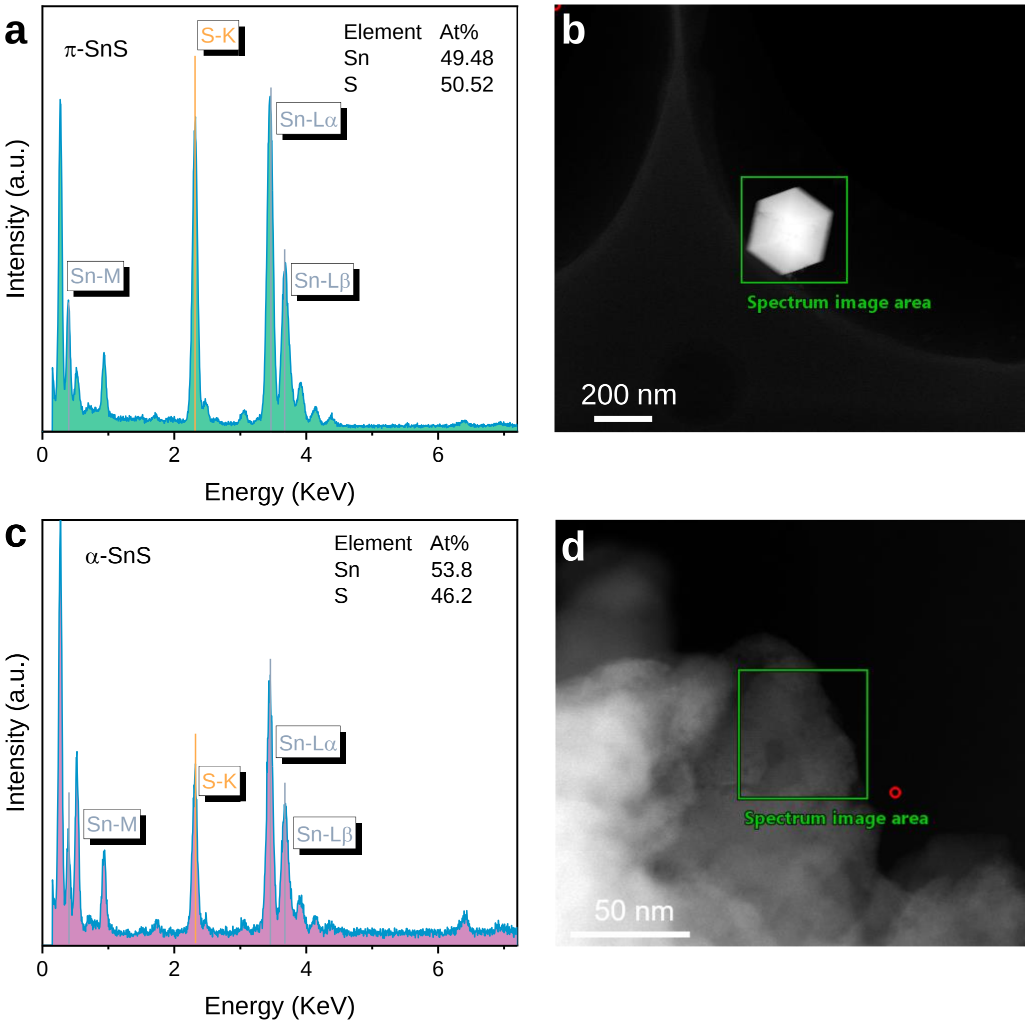

Supplementary Fig. 3 | EDX spectra and element compositions of (**a**) π-SnS with corresponding spectrum image area (**b**), and (**c**) α-SnS with corresponding spectrum image area (**d**).

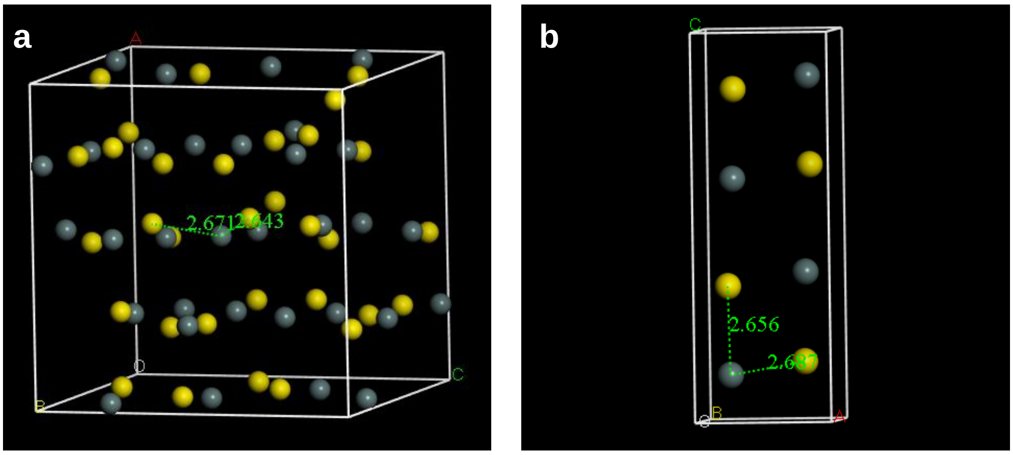

Supplementary Fig. 4 | Bond length of optimized (**a**) π-SnS and (**b**) α-SnS crystal structures.

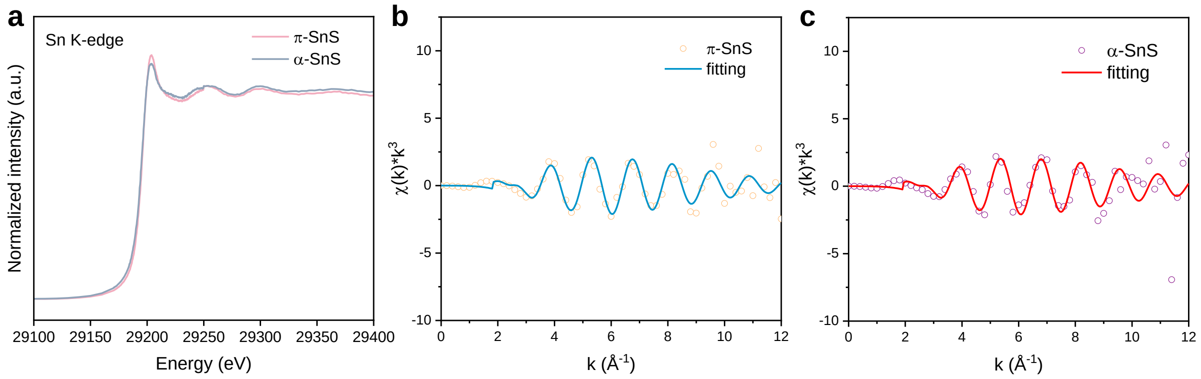

Supplementary Fig. 5 | Sn K-edge XANES spectra of π-SnS and α-SnS (**a**). K-space EXAFS of π-SnS (**b**) and α-SnS (**c**).

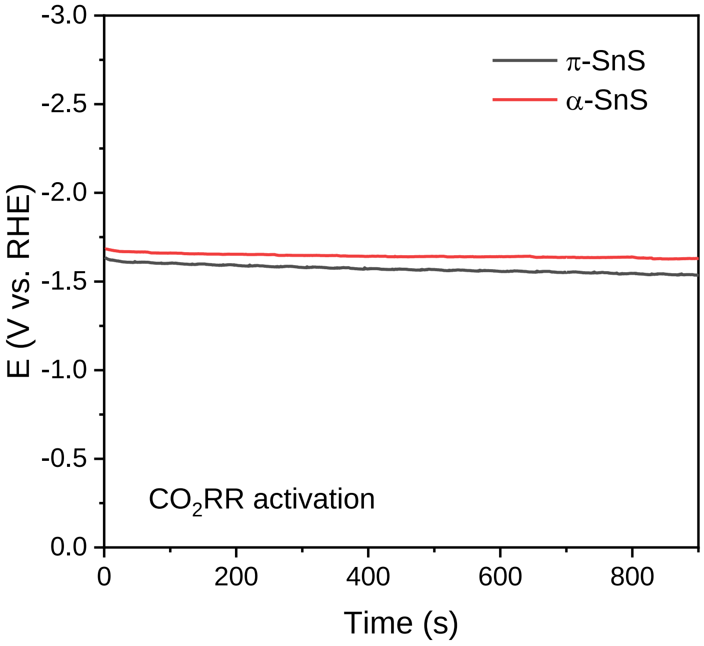

Supplementary Fig. 6 | IR compensated applied potential (E) vs. time for CO_2RR activation of π-SnS and Sn(S)-L with current density of −100 mA cm^{-2}.

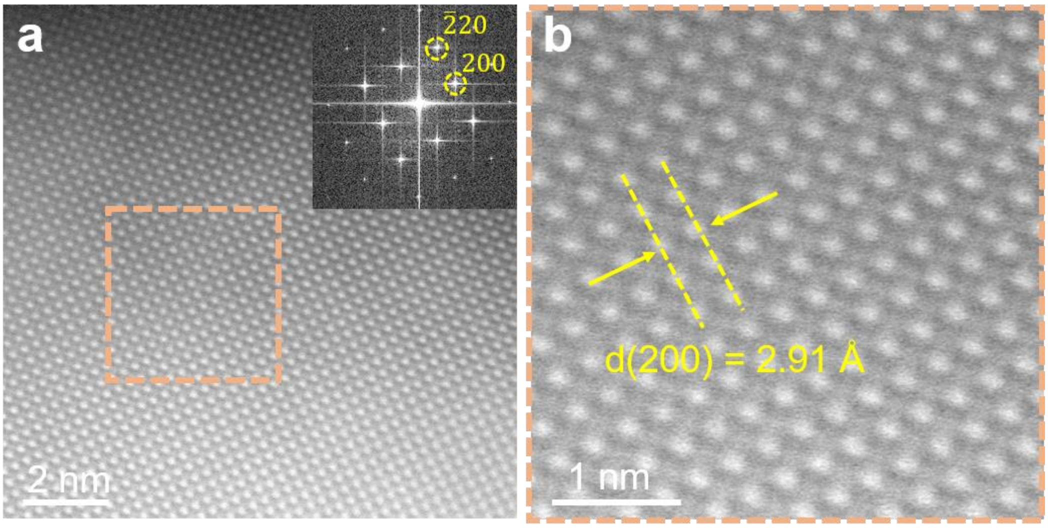

Supplementary Fig. 7 | a, HAADF-STEM image of Sn(S)-L with the inset of FFT pattern. **b**, Magnified HAADF-STEM image taken from the corresponding area in (**a**).

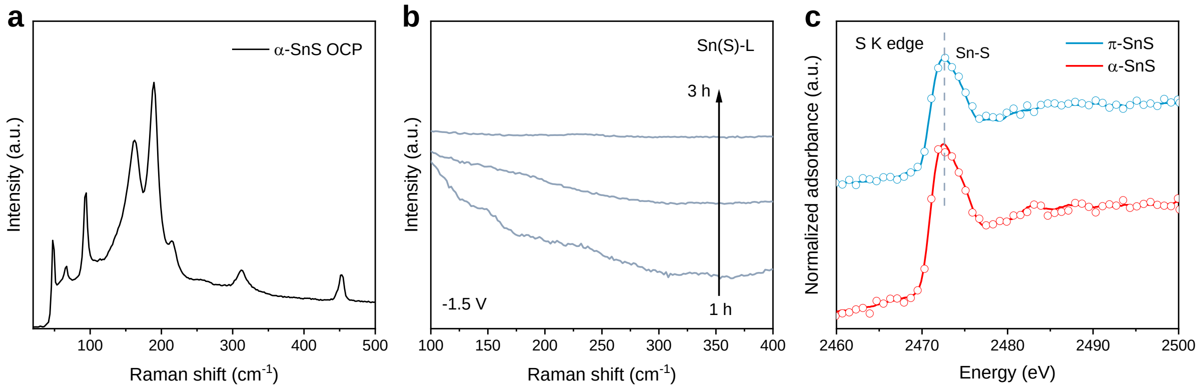

Supplementary Fig. 8 | a, In situ Raman spectra of α-SnS at open circuit potential (OCP) in CO_2-saturated K_2SO_4 + H_2SO_4 electrolyte (pH=3). **b**, In situ Raman spectra of Sn(S)-L with increasing reduction time at −1.5 V (vs. RHE) in CO_2-saturated 0.5 M K_2SO_4 solution (pH = 3). **c**, S K edge NEXAFS spectra of π-SnS and α-SnS.

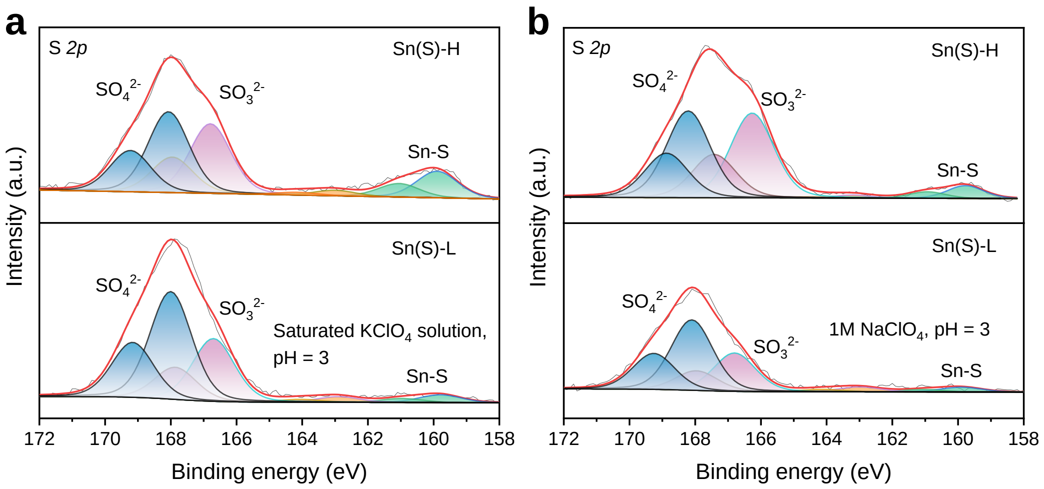

Supplementary Fig. 9 | High-resolution S *2p* XPS spectra of Sn(S)-H and Sn(S)-L after CO_2RR activation in (**a**) saturated $KClO_4$ + $HClO_4$ electrolyte and (**b**) 1 M $NaClO_4$ + $HClO_4$ electrolyte.

Note: We use the electrolyte without sulfur element to eliminate the influence of electrolyte on Sn-S detection. Potassium perchlorate ($KClO_4$) + perchloric acid ($HClO_4$) is a suitable choice. However, due to the low solubility of $KClO_4$, we use saturated potassium perchlorate (around 0.12 M) + perchloric acid (pH = 3) solution and 1 M sodium perchlorate + perchloric acid (pH = 3) solution for comparison. π-SnS derived catalysts (Sn(S)-H) illustrate higher residual S amount than α-SnS derived catalysts (Sn(S)-L) in both saturated $KClO_4$ + $HClO_4$ electrolyte and 1 M $NaClO_4$ + $HClO_4$ electrolyte, confirming the stronger Sn-S binding strength on π-SnS phase than α-SnS. The results are in accordance with that in 0.5 M K_2SO_4 + H_2SO_4 electrolyte. However, we can still find residual sulfates (SO_4^{2-}) and sulfites (SO_3^{2-}) in Sn(S)-H and Sn(S)-L. The SO_4^{2-} and SO_3^{2-} detected in **Supplementary Fig. 9** could originate from two pathways: 1) Nafion binder with high valence state of S (+6); 2) the oxidation of dissociated S (from pre-catalysts) after CO_2RR activation.

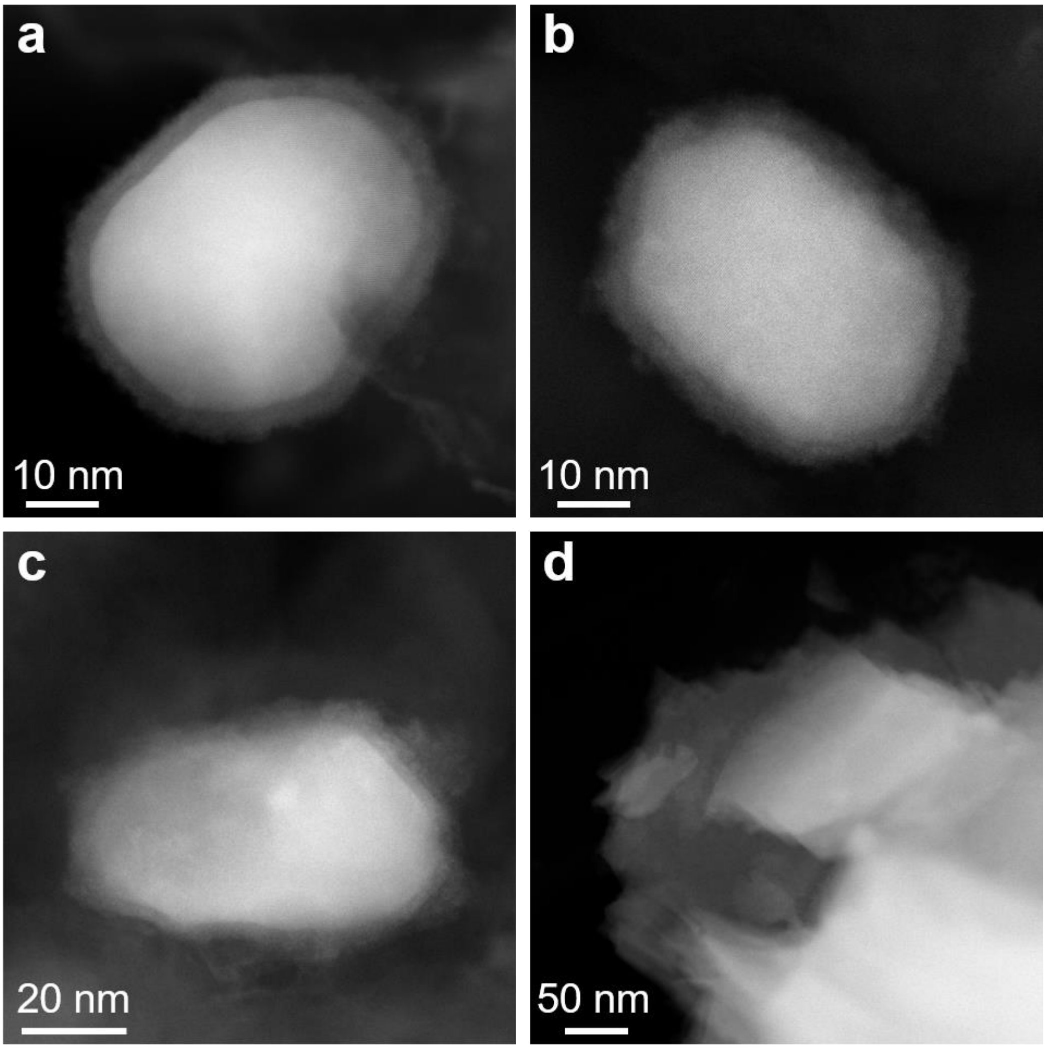

Supplementary Fig. 10 | HADDF-STEM images of Sn(S)-H (**a**,**b**) and Sn(S)-L (**c**,**d**).

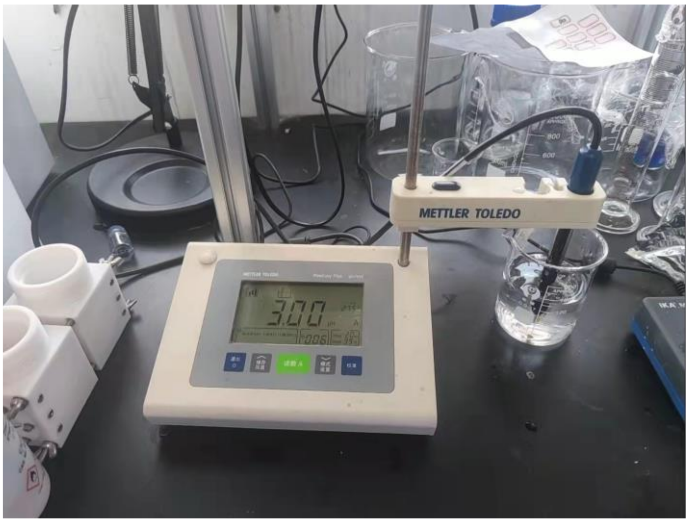

Supplementary Fig. 11 | Photograph for pH measurement of as-prepared 0.5 M K_2SO_4 + H_2SO_4 electrolyte (pH = 3).

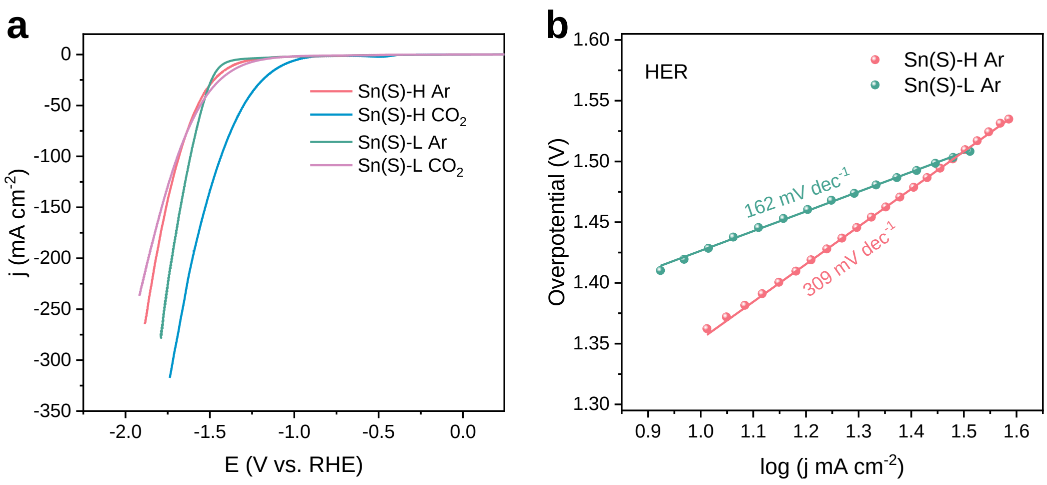

Supplementary Fig. 12 | a, IR compensated LSV curves of Sn(S)-H and Sn(S)-L collected in 0.5 M K_2SO_4 + H_2SO_4 electrolyte (pH = 3) with a scan rate of 0.05 V s^{-1}, the flow rate of Ar and CO_2 is 80 sccm. **b**, Tafel plot of HER performance for Sn(S)-H and Sn(S)-L from Ar-saturated LSV curves in (**a**).

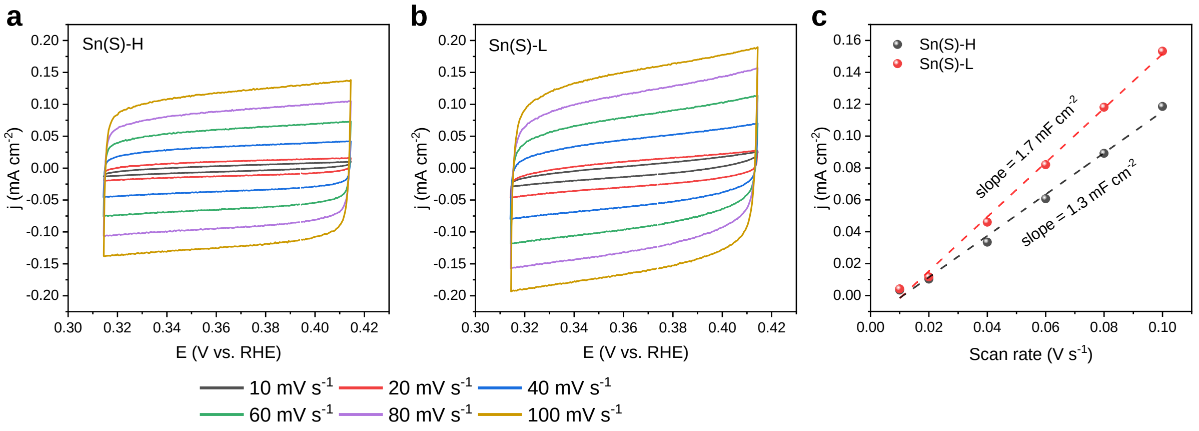

Supplementary Fig. 13 | ECSA measurement. CV curves at different scan rates in the non-Faradaic capacitance range of (**a**) Sn(S)-H and (**b**) Sn(S)-L. **c**, Current density plotted against scan rate of Sn(S)-H and Sn(S)-L.

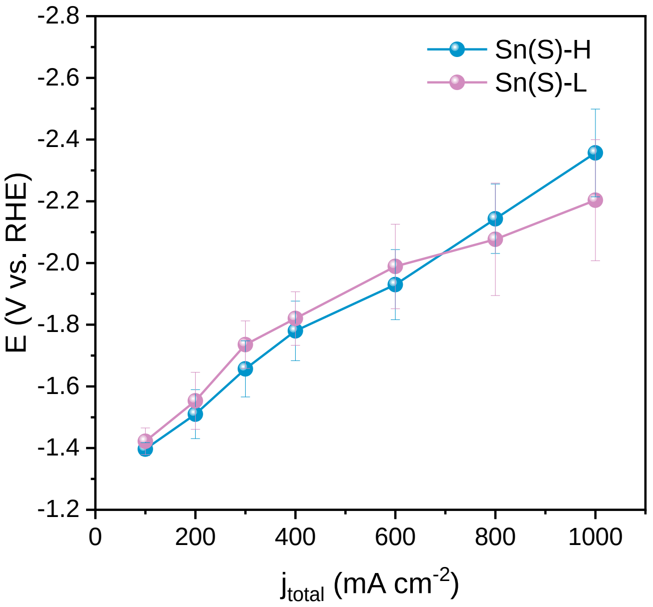

Supplementary Fig. 14 | Current density-dependent potential (IR compensated) of Sn(S)-H and Sn(S)-L. Error bars correspond to the standard deviation of three independent measurements.

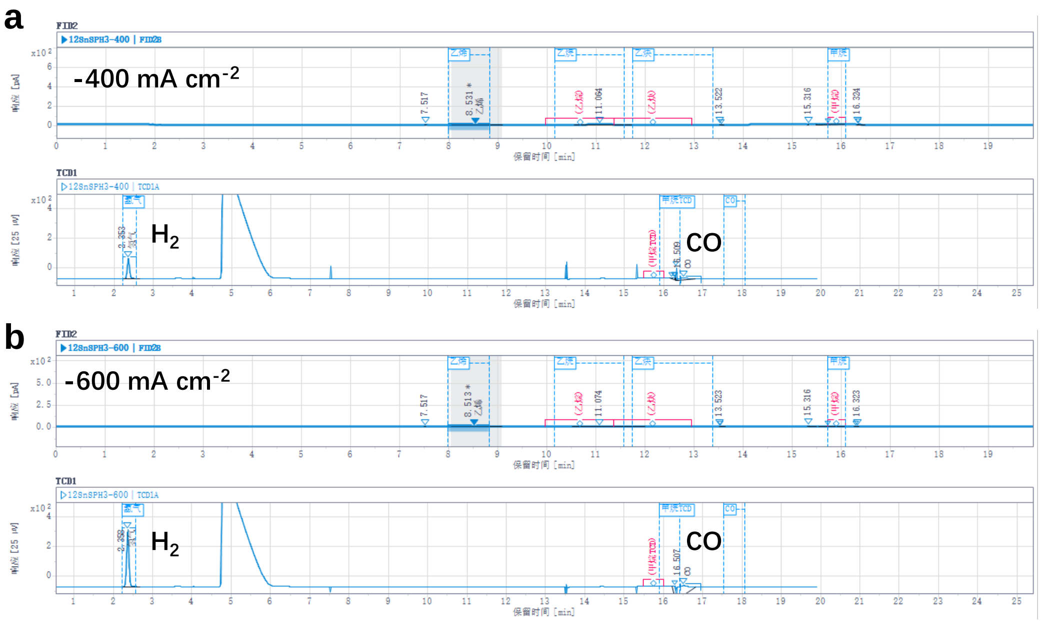

Supplementary Fig. 15 | GC trace of gaseous products (H_2 and CO) during electrochemical CO_2RR of Sn(S)-H at (a) −400 mA cm^{-2} and (b) −600 mA cm^{-2}.

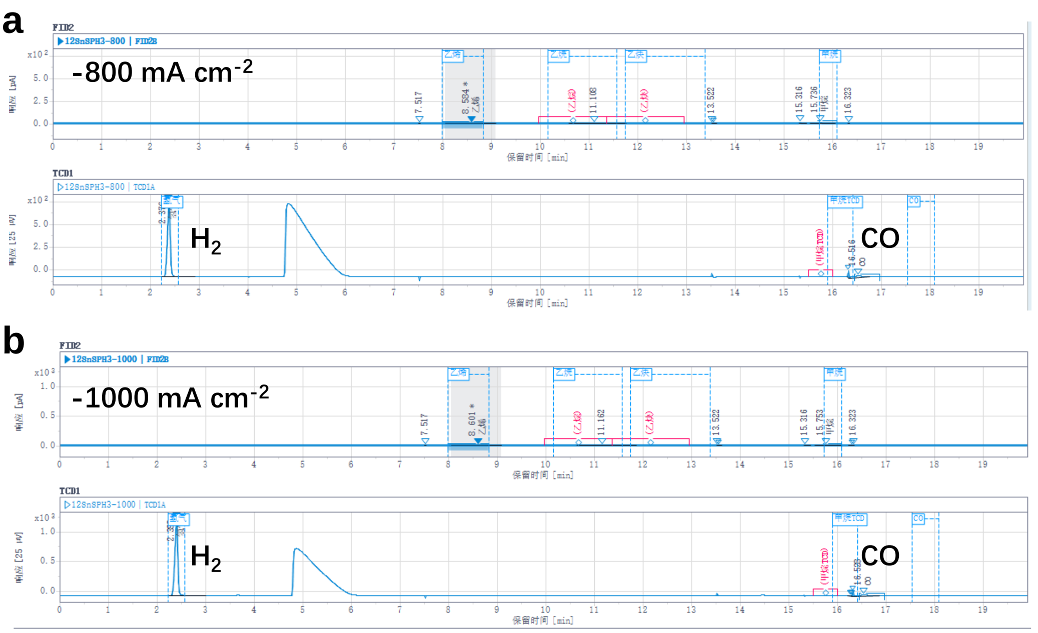

Supplementary Fig. 16 | GC trace of gaseous products (H_2 and CO) during electrochemical CO_2RR of Sn(S)-H at (a) −800 mA cm^{-2} and (b) −1000 mA cm^{-2}.

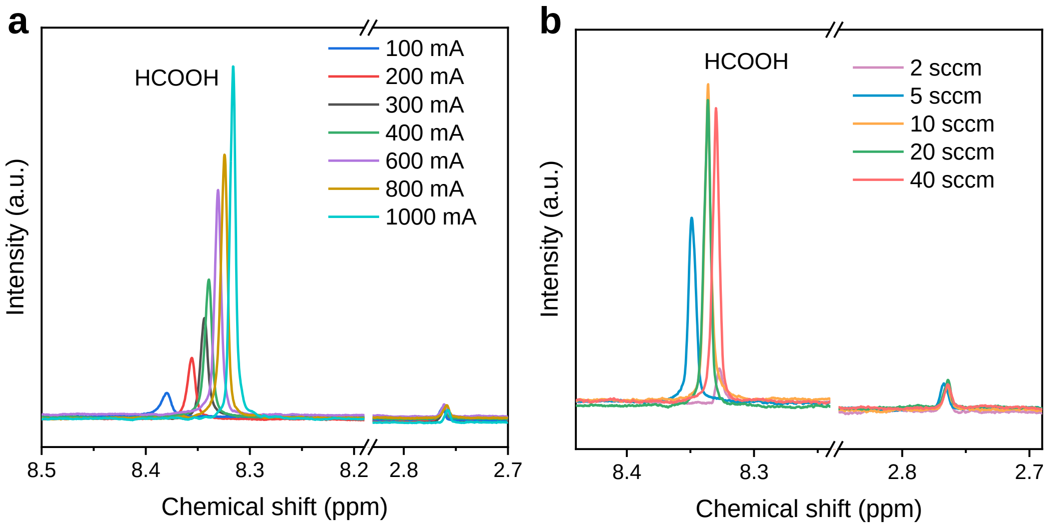

Supplementary Fig. 17 | ^{1}H-NMR spectrum. **a**, HCOOH products spectrum form SnS-H in CO_2RR under different current densities. **b**, HCOOH products spectrum form SnS-H in CO_2RR with different CO_2 flow rates (−400 mA cm^{-2}).

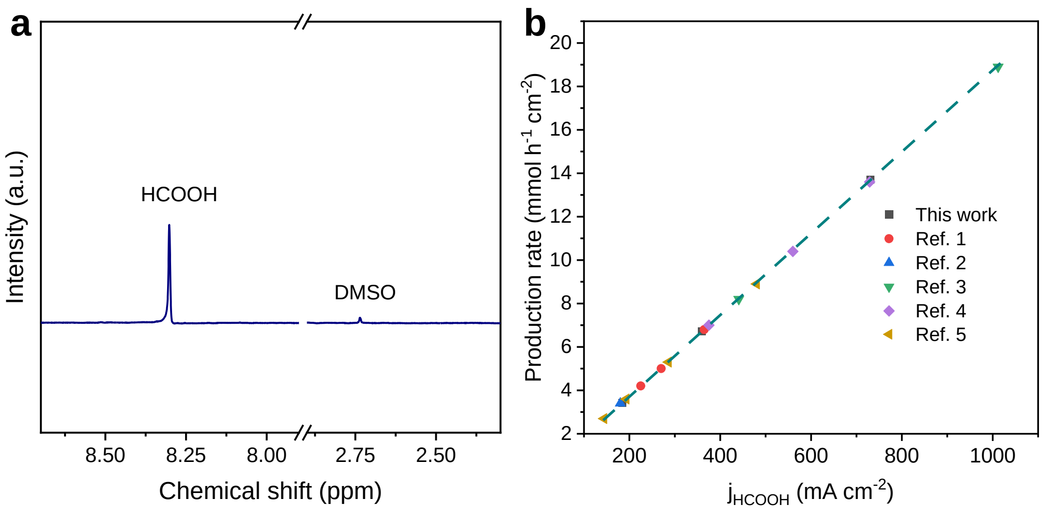

Supplementary Fig. 18 | Detection of the linear relationship of particle HCOOH current densities (j_{HCOOH}) and production rate. **a**, Selected ^{1}H-NMR of produced HCOOH solution. **b**, Linear curve of reported j_{HCOOH} against HCOOH production rate.

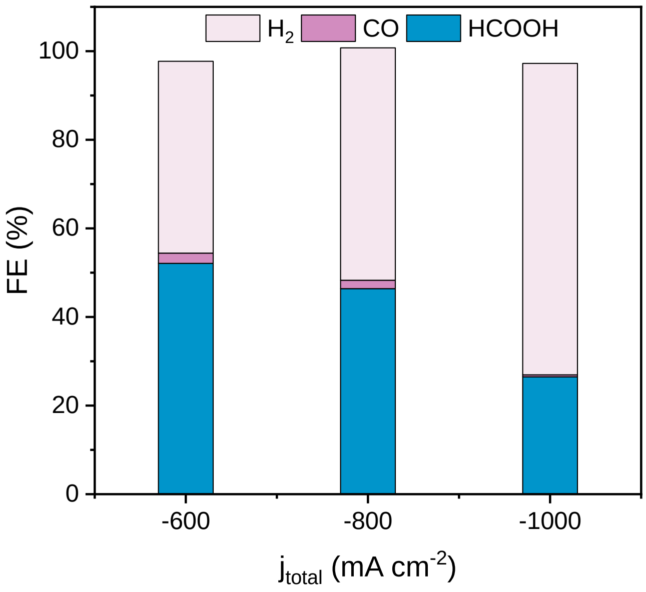

Supplementary Fig. 19 | FE value of CO_2RR products for pure metallic Sn at high current densities.

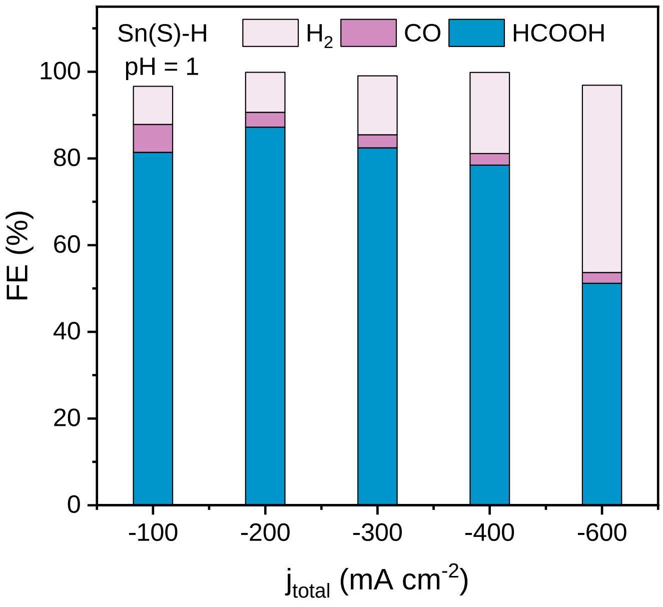

Supplementary Fig. 20 | FE value of CO_2RR products for Sn(S)-H under selected current densities at pH = 1.

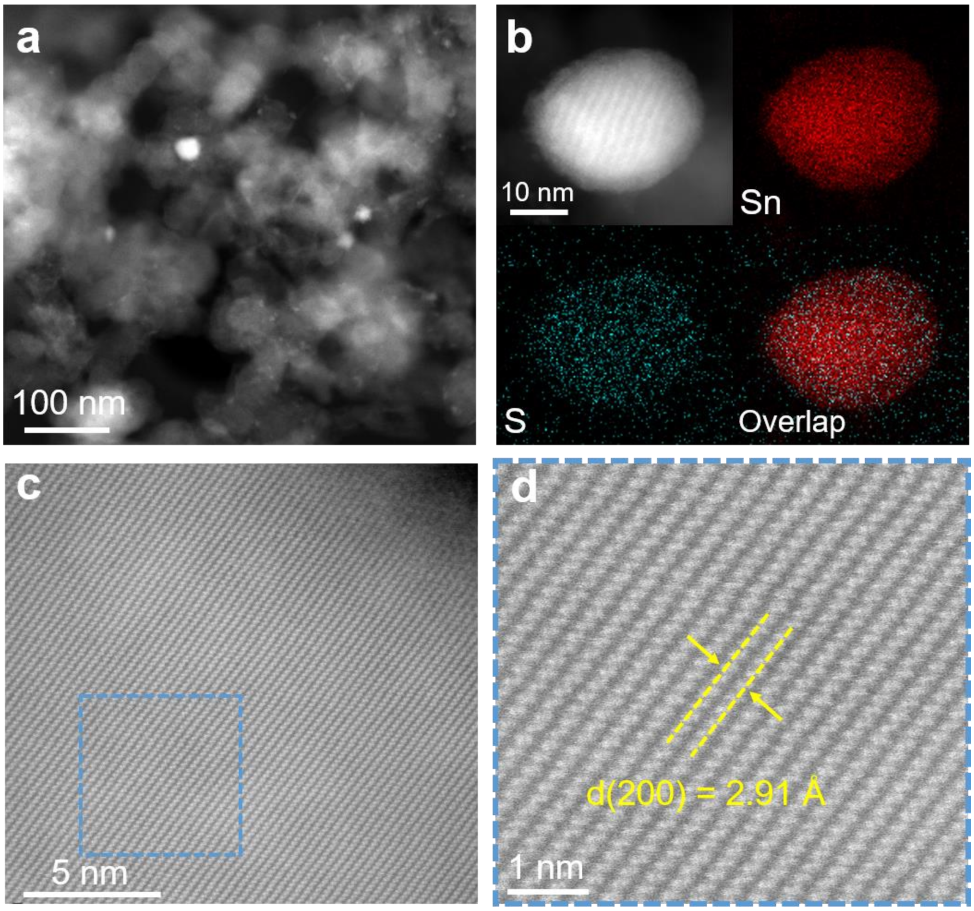

Supplementary Fig. 21 | Structural characterization of Sn(S)-H after stability measurement. **a**, TEM image. **b**, EDS mapping images of Sn and S elements. **c**, HAADF-STEM image. **d**, Magnified HAADF-STEM image taken from the corresponding area in (**c**).

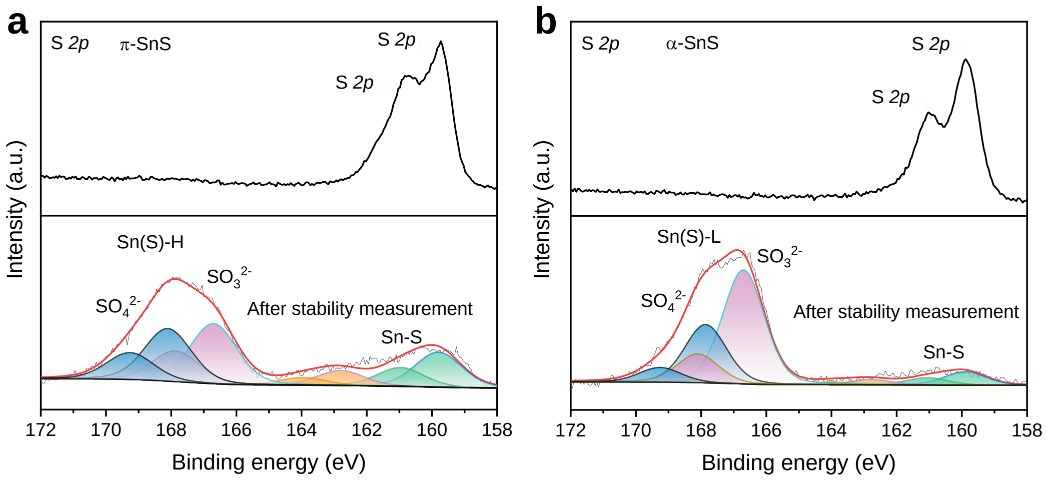

Supplementary Fig. 22 | High-resolution S *2p* XPS spectra of (**a**) Sn(S)-H and (**b**) Sn(S)-L after stability measurement with the corresponding pre-catalysts π-SnS (**a**) and α-SnS (**b**) inserted in top.

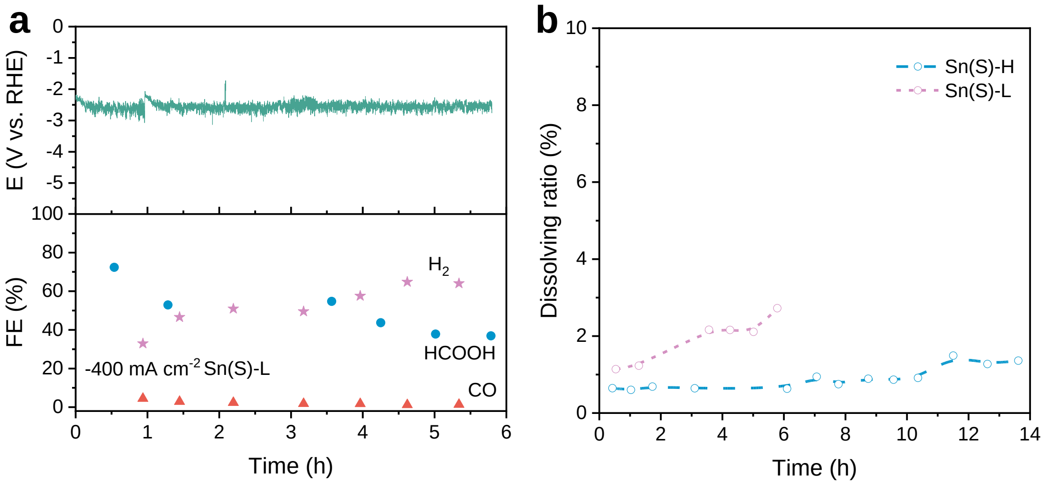

Supplementary Fig. 23 | a, Stability measurement of HCOOH production under the total current density of −400 mA cm^{-2} for Sn(S)-L. **b**, Dissolving ratio of Sn(S)-H and Sn(S)-L at different reaction time during the stability measurement.

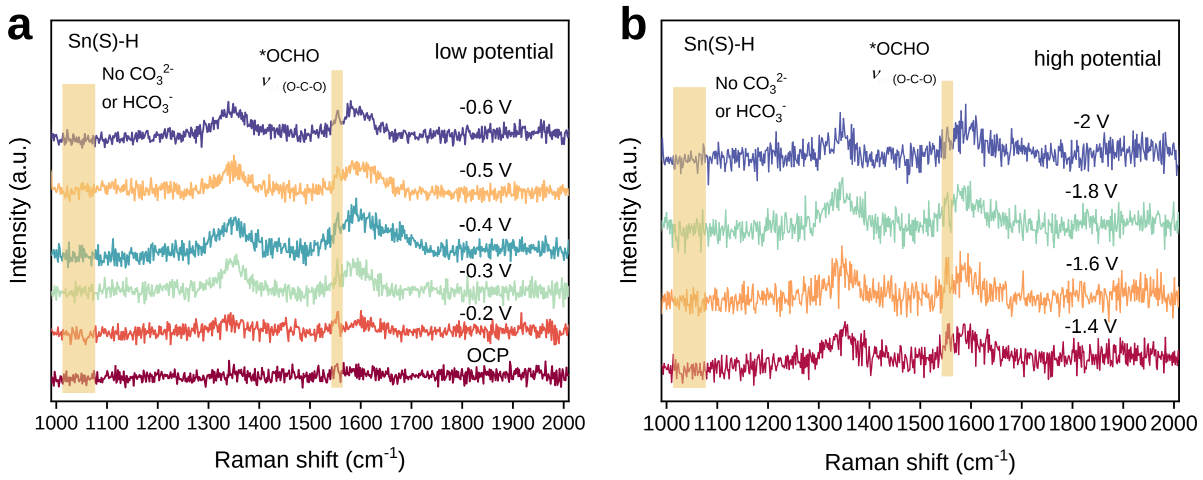

Supplementary Fig. 24 | In situ SERS of Sn(S)-H under CO_2RR with increased potential in flow cell. **a**, Low potential (OCP to –0.6 V). **b**, High potential (−1.4 to −2 V).

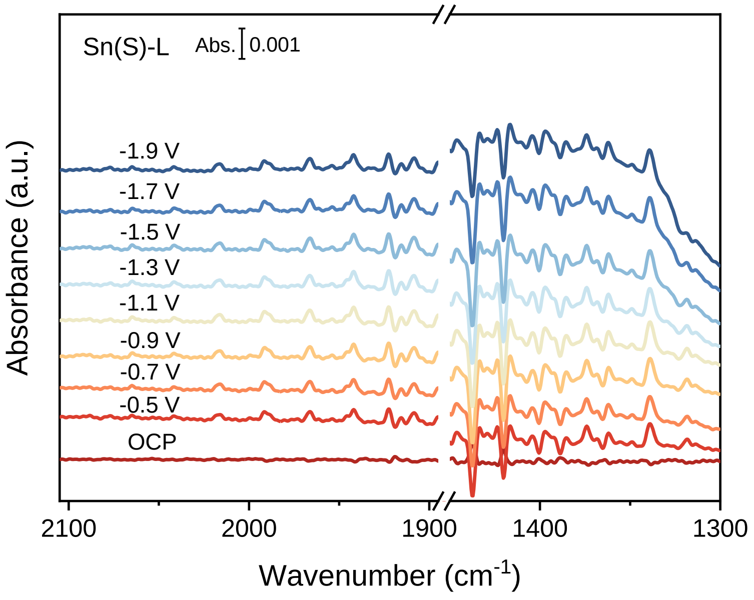

Supplementary Fig. 25 | In situ ATR-FTIR spectra of Sn(S)-L.

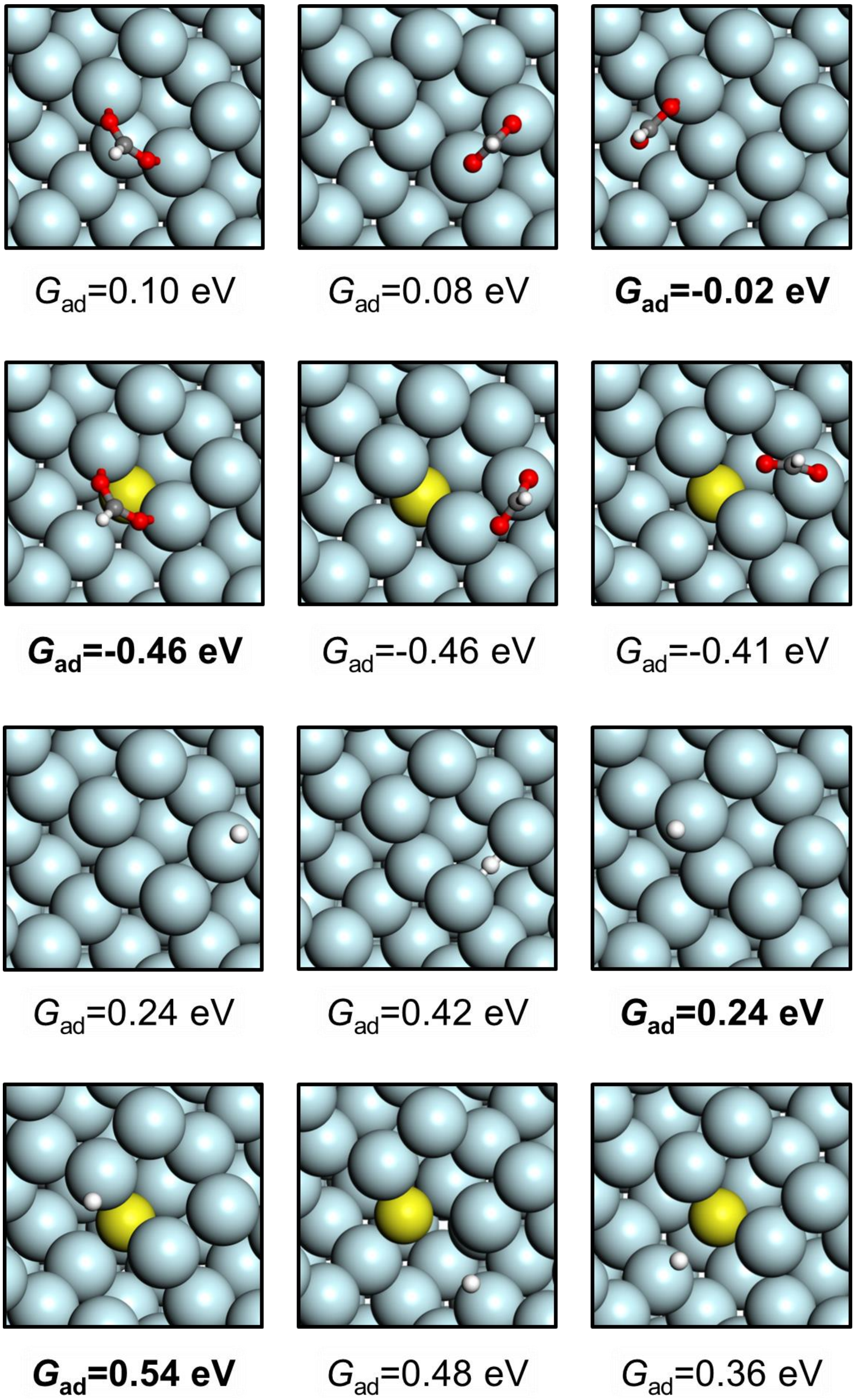

Supplementary Fig. 26 | Adsorption configuration and free energy (G_{ad}) of *OCHO and *H on Sn (100) and S-doped Sn (100). The adsorption site and G_{ad} shown in **Fig. 4d** is marked in bold. Steel blue sphere: Sn; yellow sphere: S; grey sphere: C; red sphere: O; white sphere: H.

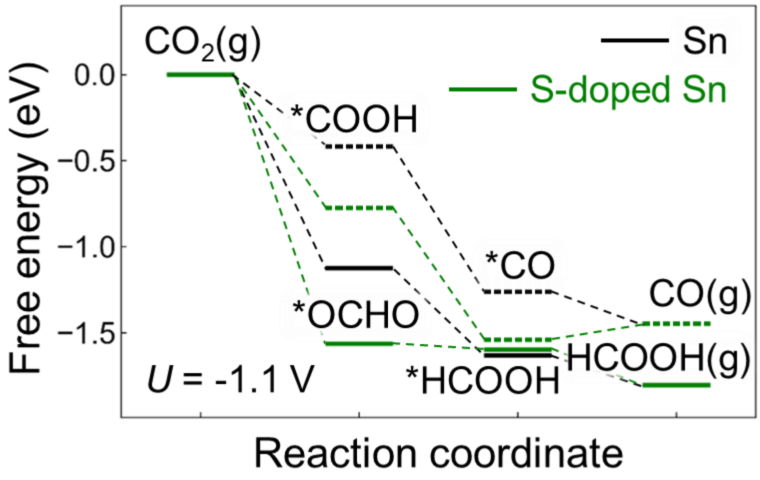

Supplementary Fig. 27 | Free energy diagram of CO_2 electroreduction to HCOOH (solid steps, CO_2→*OCHO→*COOH→HCOOH), compared with to CO (dashed steps, CO_2→*COOH→*CO→CO), under U = −1.1 V (vs. SHE) on Sn (100) and S-doped Sn (100). The HCOOH pathway (*via* *OCHO) is significantly more favorable than the CO pathway (*via* *COOH).

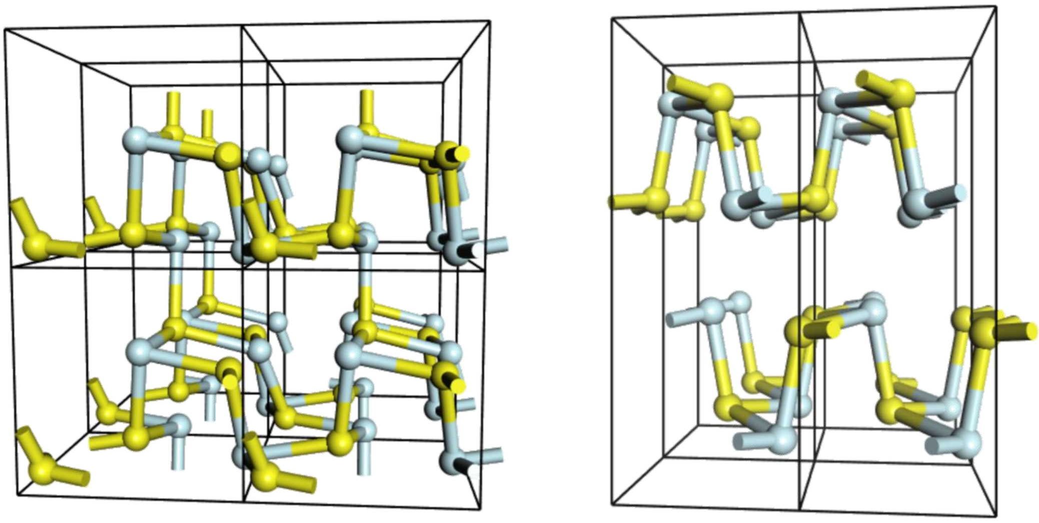

Supplementary Fig. 28 | Crystal structures for calculation of cubic-(π) and orthorhombic-(α) phase of SnS. Steel blue sphere: Sn; yellow sphere: S.

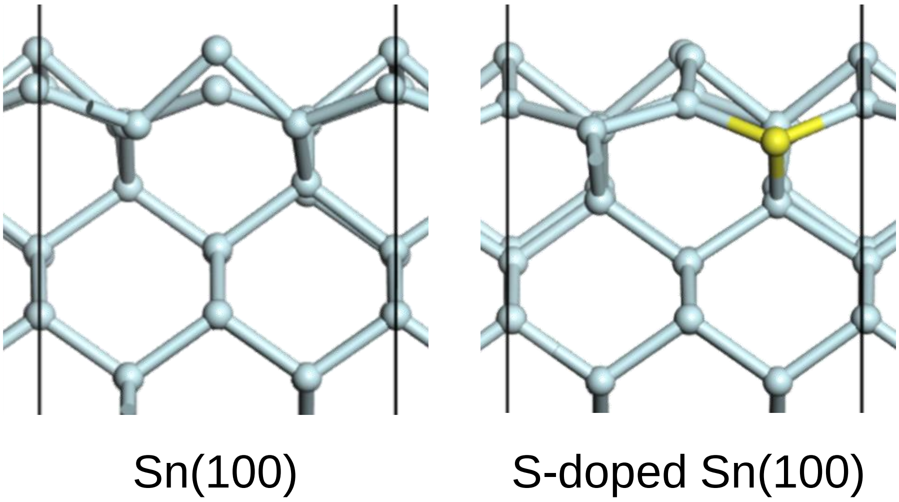

Supplementary Fig. 29 | Surface structures for calculation of Sn and S-doped Sn (in subsurface). Steel blue sphere: Sn; yellow sphere: S.

Supplementary Table 1 | Performance metrics of different CO_2RR formate/formic acid generation catalysts in flow cell

Catalyst	Electrolyte	pH	FE	j_{HCOOH} (mA·J cm^{-2})	r_{HCOOH} (mmol·h^{-1}·cm^{-2})	Ref.
π-SnS	0.5 M K_2SO_4 + H_2SO_4	3	92.15%	184.3	3.4	This work
			89.8%	359.2	6.8	
			73%	730.2	13.7	
SnO_2/C	0.1 M H_2SO_4 + 0.4 M K_2SO_4	1.5	88%	218	4.1	Nat. Catal. 2022, 5, 268.
			76%	314	5.8	
Bi nanosheets	0.05 M H_2SO_4 + 3 M KCl	0.5	92.2%	273.1	5.1	ACS Catal. 2022, 12, 2357.
$Sn_{0.80}Bi_{0.20}$@Bi-SnO_x	0.5 M $KHCO_3$	7.2	95.8%	20.9	0.4	Adv. Mater. 2020, 32, 2002822.
			72.1%	74.6	1.4	
Sn-Cu/SnO_x core-shell NPs	1 M KOH	13.5	90%	218.8	4.2	Angew. Chem. Int. Ed. 2020, 59, 4814.
			88%	357.9	6.8	
POD-Bi	1 M KOH	13.5	93%	93	1.7	Angew. Chem. Int. Ed. 2018, 57, 16114.
Bismuth oxide nanotubes	1 M KOH	13.5	97%	279.3	5.2	Nat. Comm. 2019, 10, 1.
	1 M $KHCO_3$	8.5	95%	136	2.5	
MIL-68(In)-NH_2	1 M $KHCO_3$	8.5	94.4%	108	2	Angew. Chem. Int. Ed. 2021, 60, 19107.
Bi_2O_3@C	1 M KOH	13.5	93%	208	3.9	Angew. Chem. Int. Ed. 2020, 132, 10899.
In_2O_3@C	1 M KOH	13.5	93.5%	138	2.6	Angew. Chem. Int. Ed. 2022, e202200552

BiOBr templated catalyst	2 M $KHCO_3$	8.1	90%	180	3.4	Adv. Mater. 2018, 30, 1802858.
nBuLi-Bi	1 M $KHCO_3$	8.5	96%<br>92%	193<br>460	3.6<br>8.5	Nat. Comm. 2020, 11, 3633.
$Bi_{0.1}Sn$	1 M KOH	13.5	95%	285	5.4	Nat. Comm. 2021, 12, 5223.

References

1. Ye, K. et al. In situ reconstruction of a hierarchical Sn-Cu/SnO_x core/shell catalyst for high-performance CO_2 electroreduction. *Angew. Chem. Int. Ed.* **59**, 4814-4821 (2020).
2. Garcia de Arquer, F. P. et al. 2D metal oxyhalide-derived catalysts for efficient CO_2 electroreduction. *Adv. Mater.* **30**, e1802858 (2018).
3. Yan, S. et al. Electron localization and lattice strain induced by surface lithium doping enable ampere-level electrosynthesis of formate from CO_2. *Angew. Chem. Int. Ed.* **60**, 25741-25745 (2021).
4. Grigioni, I. et al. CO_2 electroreduction to formate at a partial current density of 930 mA cm^{-2} with InP colloidal quantum dot derived catalysts. *ACS Energy Lett.* **6**, 79-84 (2020).
5. Fan, L., Xia, C., Zhu, P., Lu, Y. & Wang, H. Electrochemical CO_2 reduction to high-concentration pure formic acid solutions in an all-solid-state reactor. *Nat. Commun.* **11**, 3633 (2020).
